# Proton pump inhibitor use and long-term outcomes in SSc: a real-life analysis from the EUSTAR database

**DOI:** 10.1093/rheumatology/keag521

**Published:** 2026-09-18

**Authors:** Francesco Bonomi, Giulia Bandini, Corrado Campochiaro, Veronica De Silvestri, Svetlana Agachi, Christina Bergmann, Oliver Distler, Brigitte Granel, Joerg Henes, Florenzo Iannone, Luca Idolazzi, Eduardo Kerzberg, Mohammad E Naffaa, Hadi Poormoghim, Mislav Radic, Marie-Elise Truchetet, Silvia Bellando Randone, Marco Matucci Cerinic, Zsuzsanna H McMahan, Michael Hughes, EUSTAR Group, Ulrich Andreas Walker, Radim Bečvář, Elise Siegert, Yannick Allanore, Carlomaurizio Montecucco, Murat Inanc, Patricia Carreira, Gábor Kumánovics, Michele Iudici, Przemyslaw Kotyla, Elisabetta Zanatta, Gianluca Moroncini, Paolo Airò, Alexandra Balbir-Gurman, Annamaria Iagnocco, Josko Mitrovic, Christopher Denton, Madelon Vonk, Gianluigi Bajocchi, Tânia Santiago, Maria De Santis, David Launay, Valeria Riccieri, Andra Balanescu, Ana Maria Gheorghiu, Vanessa Smith, Addolorata Corrado, Mette Mogensen, Marie Vanthuyne, Juan Jose Alegre Sancho, Branimir Anić, Carolina de Souza Müller, Alberto Cauli, Kamal Solanki, Eiman Soliman, Edoardo Rosato, Britta Maurer, Sophie Blaise, Patricia Senet, Emmanuel Chatelus, Francesco Del Galdo, Ivan Castellví, Massimiliano Limonta, Antonella Marcoccia, Ina Koetter, Vivien Hsu, Dinesh Khanna, Carolina Teles, Yair Levy, Elena Rezus, Daniel Brito de Araujo, Giovanna Cuomo, Petros Sfikakis, Ana-Maria Ramazan, Jeska de Vries-Bouwstra, Alain Lescoat, Julia Spierings, Fabiola Atzeni, Masataka Kuwana, Mickaël Martin, Alfredo Guillén- Del-Castillo, Magda Parvu, Gonçalo Boleto, Nicoletta Del Papa, Enrico Selvi, Marta Mosca, Ulrich Gerth, Duygu Temiz Karadag, Anastas Batalov, Cristina Maglio, Miriam Retuerto, Monique Hinchcliff, Samar Tharwat, Ana Cristina Cordeiro, Francesco Benvenuti, Andrea-Hermina Györfi, Lilian Maria Lopez Nunez, Rossella De Angelis, Alejandro Brigante, Yasser El Miedany, Amato de Paulis, Chris Derk, Lijun Zhang, Bogdan Batko, Ivette Casafont Sole, Anna Lewandowska-Polak, Belén Atienza-Mateo, Tuncay Duruöz, Seda Colak, Janeth Villegas Guzmán, Claudia Mora-Trujillo, Boris Lindner, Maria Sole Chimenti, Samah A El-Bakry, Vera Ortiz-Santamaria, Jorge Rojas-Serran, Fatma Alibaz-Oner, Emine Duygu Ersözlü, Roberto Muñoz Louis, Minrui Liang, Massimo Triggiani, Anastasios Karamanakos, Bianca Dumitru, Lucy Carter, Beatrice André, Giuseppe Murdaca, Patricia Corzo Garcia, Theodora Simopoulou, Fabio Cacciapaglia, Tejaswini Ramineni, Gaia Mansuco, Jhonny Calizaya-Estrada, Gehad Gamal Maghraby, Mustafa Erdogan, Wen-Nan Huang, Graça Sequeira, Greta Pellegrino, Cláudia Vaz, Maya Buch, Joanna Kosałka-Węgiel

**Affiliations:** Department of Internal Medicine, University Hospital Careggi, Florence, Italy; Department of Internal Medicine, University Hospital Careggi, Florence, Italy; Unit of Immunology, Rheumatology, Allergy and Rare Diseases (UnIRAR), IRCCS San Raffaele Scientific Institute, Milan, Italy; Department of Internal Medicine, University Hospital Careggi, Florence, Italy; Discipline of Rheumatology and Nephrology, Department of Internal Medicine, Nicolae Testemițanu State University of Medicine and Pharmacy, Chisinau, Moldova; Department of Internal Medicine 3-Rheumatology and Immunology, Friedrich-Alexander-Universität (FAU) Erlangen-Nürnberg and Universitätsklinikum Erlangen, Erlangen, Germany; Deutsches Zentrum Immuntherapie (DZI), FAU Erlangen-Nürnberg and Universitätsklinikum Erlangen, Erlangen, Germany; Department of Rheumatology, University Hospital Zurich, University of Zurich, Zurich, Switzerland; Department of Internal Medicine, Hôpital Nord, Assistance Publique-Hôpitaux de Marseille (AP-HM), Marseille, France; Center for Interdisciplinary Rheumatology, Immunology and Autoimmune Diseases (INDIRA) and Department for Internal Medicine II (Hematology, Oncology, Rheumatology and Clinical Immunology), University Hospital Tuebingen, Tuebingen, Germany; Department of Precision and Regenerative Medicine and Ionian Area (DiMePRe-J), Polyclinic Hospital, University of Bari, Bari, Italy; Rheumatology Section, Department of Medicine, University of Verona, Verona, Italy; Departamento de Medicina Interna, Hospital General de Agudos J.M Ramos Mejía, Buenos Aires, Argentina; The Rheumatology Unit, Galilee Medical Center, Nahariyya, Israel; The Azrieli’s Faculty of Medicine, Bar-Ilan University, Safed, Israel; Iran University of Medical Sciences, Tehran, Iran; Department of Internal Medicine, Division of Rheumatology, Allergology and Clinical Immunology, Center of Excellence for Systemic Sclerosis in Croatia, University Hospital of Split, Split, Croatia; Internal Medicine Department, School of Medicine, University of Split, Split, Croatia; ImmunoConcEpT, UMR 5164, University Bordeaux, CNRS, Bordeaux, France; CHU de Bordeaux, FHU ACRONIM, Centre national de référence des maladies auto-immunes et systémiques rares Est/Sud-Ouest (RESO), Bordeaux, France; Department of Experimental and Clinical Medicine, Division of Rheumatology, Careggi University Hospital, University of Florence, Florence, Italy; Unit of Immunology, Rheumatology, Allergy and Rare Diseases (UnIRAR), IRCCS San Raffaele Scientific Institute, Milan, Italy; Vita Salute San Raffaele University, Milano, Italy; Inflammation Fibrosis and Ageing Initiative, (INFLAGE), Division of Genetics and Cell Biology, IRCCS San Raffaele Scientific Institute, Milano, Italy; Division of Rheumatology, UTHealth Houston, Houston, TX, USA; Centre for Musculoskeletal Research, Division of Musculoskeletal and Dermatological Science, School of Biological Sciences, Faculty of Biology Medicine and Health, The University of Manchester, Manchester Academic Health Science Centre, Manchester, UK; Northern Care Alliance NHS Foundation Trust, Salford Care Organisation, Salford, UK; NIHR Manchester Biomedical Research Centre, Manchester University NHS Foundation Trust, Manchester, UK

**Keywords:** SSc, scleroderma, proton pump inhibitor, interstitial lung disease

## Abstract

**Objectives:**

To investigate the association between proton pump inhibitor (PPI) use, all-cause mortality and interstitial lung disease (ILD) progression in SSc.

**Methods:**

SSc patients enrolled in the EUSTAR registry with at least two clinical visits were included (EUSTAR CP141). PPI exposure was modelled as a time-varying variable. Inverse probability of treatment weighting (IPTW) based on propensity scores was applied to address confounding by indication. All-cause mortality was analysed using IPTW-weighted time-varying Cox models. ILD progression was assessed using functional endpoints (FVC decline ≥10% and ≥5%; DLCO decline ≥10%). Restricted mean survival time (RMST) was used to quantify absolute effects. Sensitivity analyses included a 6-month lag time.

**Results:**

Out of 10 660 patients enrolled, 8637 (81.0%) were exposed to PPIs during a mean follow-up of 5.7 ± 4.3 years. They exhibited a more severe multisystem disease phenotype, including greater gastrointestinal, pulmonary and vascular involvement. After adjustment, PPI exposure was associated with higher all-cause mortality (HR 1.88, 95% CI 1.40–2.54), although the RMST difference at 5 years was minimal. As cause-specific mortality was unavailable, this association might reflect underlying disease severity rather than a direct drug effect. The use of PPI was not associated with a reduced risk of clinically meaningful FVC decline, while a modest attenuation of DLCO decline was observed, and absolute progression-free differences were limited.

**Conclusions:**

PPI is commonly used in SSc characterized by a more severe multisystem phenotype. Only a marginal attenuation of ILD progression is observed, although absolute effects were small. These findings favour the generalized use of PPIs for symptomatic gastroesophageal reflux in SSc.

*Rheumatology* key messages

- PPI use identifies SSc patients with more severe multisystem disease phenotype.
- No clinically meaningful survival difference despite higher relative mortality estimates after adjustment.
- Only modest attenuation of ILD progression, with limited absolute clinical benefit.

## Introduction

Gastroesophageal reflux disease (GERD) is among the most prevalent manifestations of SSc, affecting over 90% of patients, and often representing the earliest clinical feature [1]. Beyond its impact on daily functioning, GERD complicates disease management by reducing drug tolerance, impairing quality of life and predisposing to oesophageal strictures, Schatzki rings and Barrett’s oesophagus [2]. Chronic acid exposure may also drive pulmonary microaspiration, a mechanism hypothesized to exacerbate interstitial lung disease (ILD), the leading cause of SSc-related mortality [3].

Proton pump inhibitors (PPIs) constitute the mainstay of therapy for GERD in SSc and are frequently initiated early and often maintained long-term [4]. Their widespread use has substantially improved symptom control and mucosal healing. However, profound oesophageal dysmotility and the high prevalence of weakly acidic or non-acid reflux often result in incomplete symptom control, leading to chronic high-dose or long-term PPI therapy despite uncertain efficacy [5].

In recent years, increasing attention has been directed towards the potential unfavourable consequences of prolonged acid-suppressive therapy. In the general population, long-term PPI exposure has been variably associated with adverse outcomes, including risk of infections, chronic kidney disease, osteoporosis, vascular calcification and increased mortality, although causality remains much debated [6–9]. These concerns may be particularly relevant in patients with SSc, including where vasculopathy and impaired calcium metabolism could potentially amplify susceptibility to PPI-related adverse effects [10, 11]. These concerns are underscored by recent multinational surveys (by both patients and clinicians alike) reporting widespread long-term PPI use in SSc and persistent safety concerns [12, 13].

Despite widespread, often long-term use of PPIs in SSc, robust evidence of their impact on major disease outcomes remains limited and conflicting. Nevertheless, current European Alliance of Associations for Rheumatology (EULAR) recommendations support use of PPIs for the management of GERD, despite the absence of SSc-specific studies demonstrating clear long-term efficacy or safety [14].

In this context, clarifying the association between chronic PPI therapy and major clinical outcomes in SSc is highly relevant to clinical practice. Using a large multinational cohort from the EUSTAR registry and time-varying analytical approaches designed to mitigate treatment-related biases, this study aimed to clarify the association between PPI exposure and major long-term outcomes in SSc, including overall survival and ILD progression.

## Methods

### Cohort selection

This longitudinal observational study was based on prospective data from patients enrolled in the European Scleroderma Trials and Research (EUSTAR) database (EUSTAR CP141) [15]. For the present analysis, we included adult patients fulfilling the 2013 American College of Rheumatology (ACR)/EULAR classification criteria for SSc [15], and with at least two recorded clinical visits, with no minimum follow-up requirement. Patients with very early diagnosis of SSc (VEDOSS) who did not fulfil the 2013 ACR/EULAR classification criteria were not included. The sine scleroderma subgroup was defined as patients fulfilling the 2013 ACR/EULAR SSc classification criteria without clinically evident skin thickening. Patients with only one recorded visit followed by a documented date of death were excluded, as the time-varying exposure analysis required longitudinal information on treatment status and covariates to construct valid follow-up intervals. This exclusion applied to five patients. The same eligibility criteria were applied to both PPI-exposed and non-exposed patients to ensure comparable follow-up structures across groups.

### Exposure definition

Exposure to PPI was modelled as a time-varying variable. Given the high frequency of treatment initiation, discontinuation and re-initiation during follow-up, static exposure definitions (e.g. ever/never use) were considered inappropriate for inferential analyses and potentially prone to immortal-time bias.

Follow-up time for each patient was therefore partitioned using a counting-process approach, generating one or more observation intervals defined by start and stop times, during which PPI exposure status remained constant. Patients could contribute person-time for exposed and unexposed states at different stages of follow-up. This approach allows estimation of the association between current PPI exposure and outcome risk at the time corresponding to exposure status. Cumulative exposure metrics were not modelled because the database does not capture the specific agent or dosage, thereby preventing a reliable reconstruction of cumulative dose or treatment intensity. For descriptive purposes, baseline characteristics were summarized according to ever- vs never-PPI exposure during follow-up.

### Clinical phenotypes assessment

To characterize disease phenotypes across PPI exposure groups, organ involvement variables were summarized using the most severe (worst-ever) value documented during follow-up. This approach was chosen to reflect cumulative disease burden rather than baseline status. Domains included cutaneous, vascular, pulmonary, cardiac, gastrointestinal GI and musculoskeletal involvement, as well as systemic inflammatory markers.

### Outcomes

The primary outcome was all-cause mortality, defined as death from any cause during follow-up.

The secondary outcome was ILD progression, evaluated in the subgroup of patients with established ILD at baseline. ILD progression was defined as the first qualifying decline in pulmonary function between two consecutive yearly visits (±3 months). Three predefined progression criteria were considered: (1) decline in forced vital capacity (FVC) ≥ 10%; (2) decline in FVC ≥5%; (3) decline in diffusing capacity for carbon monoxide (DLCO) ≥ 10% [16–18]. The event date were assigned to the follow-up visit at which the qualifying decline was first observed. Patients without progression were censored at the earliest of death or last available visit.

### Confounding control and propensity score weighting

Because PPI prescription in SSc is non-random and strongly influenced by clinical characteristics, confounding was addressed using an inverse probability of treatment weighting (IPTW) approach based on the propensity score.

For the mortality analysis, the propensity score was defined as the estimated probability of receiving PPI therapy conditional on *a priori*-selected baseline covariates. For the ILD progression analyses, propensity scores were estimated at the patient-interval level in the counting-process dataset, modelling the probability of current PPI exposure conditional on fixed baseline covariates and time-updated covariates measured at the start of each interval or at the preceding visit, as available. Full details of covariates included in the propensity score models for the mortality and ILD progression analyses are reported in Supplementary Tables S1 and S2. Prior to propensity score estimation, categorical covariates were encoded as indicator variables. Missing values in covariates used for propensity score estimation were handled using an iterative multivariable imputation procedure. The imputation model included all covariates entering the propensity score model and was run for 10 iterations with a fixed random seed to ensure reproducibility. Imputed binary indicators were rounded and constrained to 0/1. Continuous variables were imputed within the same multivariable framework and subsequently standardized. The resulting complete dataset was then used to estimate propensity scores using logistic regression.

Stabilized IPTW weights were calculated and truncated at the first and 99th percentiles to limit the influence of extreme weights and improve estimation stability. Covariate balance between the exposed and unexposed groups was assessed using standardized mean differences (SMDs) before and after weighting. To account for disease severity and potential confounding, the mortality analysis was adjusted for all variables included in the validated SCleroderma mOrtality pEustar (SCOpE) score [19].

For the ILD progression analyses, covariates were selected based on predictors of pulmonary decline in SSc-ILD identified in the literature. These included disease duration, modified Rodnan Skin Score (mRSS), baseline pulmonary function parameters (FVC and DLCO), Scl-70 antibody status, smoking exposure, presence of oesophageal symptoms and use of conventional synthetic DMARDs (csDMARDs) and/or biologic DMARDs (bDMARDs) [20–23]. In analyses using DLCO decline as the outcome, additional adjustment for pulmonary hypertension was performed. Age was initially considered for inclusion in the ILD progression models but was excluded from the final models because of multicollinearity with disease duration; after exclusion, all variance inflation factors were below the prespecified threshold.

### Statistical analysis

Descriptive statistics included means (S.D.) or medians (IQR) for continuous variables and numbers (%) for categorical variables. Group differences were evaluated using Student’s *t-*test or Mann–Whitney *U*-test for continuous variables, and *χ*² or Fisher’s exact test for categorical variables.

For the mortality analysis, survival time was defined as the interval between baseline and death or last follow-up. The association between PPI exposure and all-cause mortality was evaluated using Cox proportional hazards models with time-varying exposure, incorporating IPTW weights. Follow-up time was measured from baseline visit to death or last available observation. Survival and restricted mean survival time (RMST) analyses were performed up to a maximum follow-up of 5 years, as <10% of patients remained at risk beyond this time point. To assess the proportional hazards assumption, we evaluated potential time-dependent effects of PPI exposure by estimating hazard ratios (HRs) within predefined follow-up periods (early, middle and late) and by using graphical diagnostics in log-log survival plots. For the ILD progression analyses, time-to-event was defined as the interval from baseline to the first occurrence of the qualifying functional decline or censoring. Associations between time-varying PPI exposure and ILD progression were estimated using IPTW-weighted Cox proportional hazards models in counting-process format. Effect estimates were reported as HRs with 95% CIs. RMST analyses were performed over a fixed 5-year horizon to quantify absolute progression-free time differences between exposure groups. All tests were two-sided, with *P* < 0.05 considered as statistically significant.

### Sensitivity analyses

As a sensitivity analysis, a 6-month lag was applied to address potential reverse causation and early events associated with imminent clinical deterioration. This was implemented by excluding the first 6 months of follow-up and refitting the weighted Cox models on the remaining observations.

In addition to HRs, we estimated RMST differences over a fixed five-year horizon to provide a complementary, assumption-robust measure of the association between PPI exposure and survival.

To address the potential influence of background immunosuppressive therapy on the mortality analysis, an additional sensitivity analysis was performed by further adjusting the IPTW-weighted time-varying Cox model for baseline csDMARD and/or bDMARD use.

### Ethical approval

The EUSTAR registry operates under the Declaration of Helsinki and was approved by local ethics committees at each participating centre. Written informed consent for data collection and research use was obtained from all patients in accordance with local regulatory requirements.

## Results

### Study population

After applying inclusion criteria, 10 660 patients from the EUSTAR registry were eligible for analysis, of whom 8637 (81.0%) received PPIs at least once during follow-up and 2023 (19.0%) were never exposed to PPIs. The mean (S.D.) follow-up duration was 5.7 (±4.3) years.

The mean age was slightly higher among ever users than among never users (55.6 ± 13.5 vs 53.2 ± 14.8 years; *P* < 0.05), while sex distribution and tobacco exposure were similar between the groups (Table 1). A higher proportion of deaths was observed among ever-PPI users during follow-up (8.6% vs 4.3%, *P* < 0.05).

**Table 1:** Clinical, serological and organ-specific phenotypes across PPI exposure groups in the EUSTAR cohort.

Variable	Never users	Ever users	Missing	*P*-value
Number of patients (*n* = 10 660)	*n* = 2023	*n* = 8637		
Age (years), mean (S.D.)	53.2 (14.8)	55.6 (13.5)	0	**<0.05**
Female sex, *n* (%)	1734 (85.7)	7329 (84.9)	0	0.3476
Tobacco exposure (ever), *n* (%)	688 (36.7)	3049 (37.3)	599	0.6456
Disease duration (years), mean (S.D.)	8.0 (8.1)	8.9 (8.4)	3528	**<0.05**
Mortality, *n* (%)	88 (4.3)	747 (8.6)	0	**<0.05**
ANA positivity, *n* (%)	1888 (96.2)	8263 (97.9)	260	**<0.05**
ACA positivity, *n* (%)	868 (47.5)	3510 (43.7)	793	**<0.05**
Anti-Scl70 positivity, *n* (%)	555 (30.2)	3190 (39.3)	711	**<0.05**
Anti-RNA polymerase III positivity, *n* (%)	110 (7.6)	503 (7.9)	2881	0.6781
Anti-PMScl positivity, *n* (%)	77 (7.7)	244 (4.9)	4635	**<0.05**
Anti-U1RNP positivity, *n* (%)	77 (4.8)	392 (5.5)	1923	0.2453
Anti-SSA positivity, *n* (%)	126 (12.6)	525 (14.1)	5933	0.2519
Anti-SSB positivity, *n* (%)	27 (2.7)	112 (3.0)	5951	0.7296
Skin involvement				
Extent of skin involvement				
– Limited SSc	772 (64.0)	2697 (57.3)		
– Diffuse SSc	263 (21.8)	2385 (37.0)		
– Sine scleroderma	172 (14.3)	365 (5.7)	3006	**<0.05**
Vascular involvement				
Digital ulcers (ever), *n* (%)	474 (39.8)	3488 (54.6)	3081	**<0.05**
Telangiectasia (ever), *n* (%)	1262 (63.4)	6674 (78.2)	133	**<0.05**
Digital pitting scars (ever), *n* (%)	825 (42.3)	5118 (61.1)	335	**<0.05**
Nailfold videocapillaroscopic pattern				
– Early	278 (29.5)	948 (21.4)		
– Active	500 (53.0)	2274 (51.2)		
– Late	165 (17.5)	1217 (27.4)	5278	**<0.05**
Calcinosis (ever), *n* (%)	196 (13.8)	1109 (20.0)	3699	**<0.05**
Lung involvement				
Interstitial lung disease at HRCT (ever), *n* (%)	655 (32.4)	3387 (53.5)	2312	**<0.05**
FVC <70% (ever), *n* (%)	207 (11.1)	1871 (23.0)	648	**<0.05**
%FVC (worse), mean (S.D.)	94.3 (20.6)	87.1 (22.2)	648	**<0.05**
DLCO <60% (ever), *n* (%)	515 (28.8)	3989 (50.7)	1000	**<0.05**
%DLCO (worse), mean (%)	69.4 (18.9)	59.7 (19.1)	1000	**<0.05**
Prominent dyspnoea, *n* (%)	105 (9.1)	1287 (20.5)	3218	**<0.05**
Cardiac involvement				
Arrhythmias requiring treatment (ever), *n* (%)	40 (3.3)	396 (8.5)	4793	**<0.05**
Pericardial effusion (ever), *n* (%)	76 (7.5)	740 (12.6)	3781	**<0.05**
Diastolic dysfunction (ever), *n* (%)	423 (24.1)	3177 (40.4)	1051	**<0.05**
Right bundle branch block (ever), *n* (%)	81 (8.9)	462 (12.5)	6065	**<0.05**
Right ventricular hypertrophy (ever), *n* (%)	20 (2.2)	131 (3.5)	6041	0.0704
Pulmonary hypertension (ever), *n* (%)	132 (12.3)	1375 (23.0)	3613	**<0.05**
Ventricular arrhythmias (ever), *n* (%)	22 (2.2)	307 (7.1)	5309	**<0.05**
Conduction blocks (ever), *n* (%)	255 (15.0)	1805 (23.7)	1324	**<0.05**
%LVEF (worse), mean (S.D.)	59.2 (8.3)	58.6 (7.1)	1421	**<0.05**
sPAP (mmHg) (worse), mean (S.D.)	28.1 (16.7)	34.2 (20.3)	2032	**<0.05**
Gastrointestinal involvement				
Oesophageal symptoms (dysphagia, reflux) (ever), *n* (%)	958 (47.4)	7334 (85.1)	24	**<0.05**
Gastric symptoms (early satiety, vomiting) (ever), *n* (%)	346 (17.2)	3732 (43.4)	47	**<0.05**
Intestinal symptoms (diarrhea, bloating, constipation) (ever), *n* (%)	572 (28.4)	4194 (48.8)	49	**<0.05**
Malabsorption syndrome (ever), *n* (%)	18 (2.2)	266 (6.0)	5396	**<0.05**
Other				
Renal crisis (ever), *n* (%)	27 (1.3)	260 (3.0)	31	**<0.05**
Muscle weakness (ever), *n* (%)	329 (16.5)	2453 (28.7)	121	**<0.05**
Muscle atrophy (ever), *n* (%)	125 (6.5)	1116 (13.5)	458	**<0.05**
CRP elevation (ever), *n* (%)	320 (27.4)	2761 (43.3)	3121	**<0.05**
Proteinuria >300 mg/24 h (ever), *n* (%)	185 (10.0)	1189 (14.6)	673	**<0.05**

Prominent dyspnoea was defined as moderate-to-severe dyspnoea corresponding to NYHA functional class ≥ II. Statistically significant values (*P* < 0.05) are shown in bold.

PPI, proton pump inhibitor; ILD, interstitial lung disease; HRCT, high resolution CT; FVC, forced vital capacity; DLCO, diffusing capacity of the lung for carbon monoxide; LVEF, left ventricular ejection fraction; sPAP, systolic pulmonary arterial pressure.

Concomitant treatments at baseline also differed markedly across the two groups (Table 2). Ever-PPI users were significantly more likely to receive concomitant therapies, including csDMARDs, bDMARDs, corticosteroids, vasodilators, antiplatelet agents, oral anticoagulants, prokinetics and NSAIDs (all *P* < 0.05). The use of prokinetic agents was rare among never-PPI users: only 12 patients (0.3%) received prokinetics at baseline, and 33 patients (1.6%) at any point during follow-up.

**Table 2:** Concomitant treatments at baseline across PPI exposure groups.

Number of patients (*n* = 10 660)	Never users	Ever users	Missing	*P*-value
Concomitant treatments	*n* = 2023	*n* = 8637		
csDMARDs, *n* (%)	571 (14.6)	2811 (35.1)	0	**<0.05**
bDMARDs, *n* (%)	43 (1.1)	356 (4.5)	0	**<0.05**
Prokinetics, *n* (%)	12 (0.3)	183 (2.3)	0	**<0.05**
Vasodilating agents, *n* (%)	860 (21.9)	4306 (53.8)	0	**<0.05**
Anti-platelet agents, *n* (%)	241 (6.1)	1518 (19.0)	0	**<0.05**
Oral anti-coagulant, *n* (%)	55 (1.4)	300 (3.8)	0	**<0.05**
Corticosteroids, *n* (%)	203 (5.2)	1673 (20.9)	0	**<0.05**
NSAIDs, *n* (%)	106 (2.7)	554 (6.9)	0	**<0.05**

Distribution of baseline concomitant therapies according to PPI exposure duration. Values are presented as number (percentage). Statistically significant values (*P* < 0.05) are shown in bold.

csDMARDs, conventional synthetic DMARDs; bDMARDs, biologic DMARDs.

### Disease phenotypes associated with the different PPI exposure groups

Clinical phenotypes varied across the two groups, with ever users generally presenting with a more severe disease phenotype (summarized in Table 1). Serological profiles differed between the exposure groups. Ever users showed a slightly higher prevalence of anti-topoisomerase I antibodies (39.3% vs 30.2%; *P* < 0.05), whereas anticentromere antibodies were less frequent (43.7% vs 47.5%; *P* < 0.05). No significant differences were observed in anti-RNA polymerase III, anti-SSA, anti-SSB or U1-RNP antibody titres. A similar pattern was observed for cutaneous involvement, with diffuse cutaneous SSc markedly more frequent among ever-PPI users (37.0% vs 21.8%), while sine scleroderma was more prevalent in never users (14.3% vs 5.7%; *P* < 0.05). A similar gradient was observed for vascular manifestations, with ever users exhibiting higher rates of digital ulcers (54.6% vs 39.8%), telangiectasias (78.2% vs 63.4%), digital pitting scars (61.1% vs 42.3%), late nailfold videocapillaroscopic patterns (27.4% vs 17.5%) and calcinosis (20.0% vs 13.8%) (all *P* < 0.05).

Pulmonary involvement was substantially more prevalent and severe among ever users. ILD at high resolution CT (HRCT) was documented in 53.5% of ever users compared with 32.4% of never users (*P* < 0.05). Ever users more frequently exhibited lower worst-ever mean %FVC (87.1 ± 22.2 vs 94.3 ± 20.6) and %DLCO (59.7 ± 19.1 vs 69.4 ± 18.9) (all *P* < 0.05). Prominent dyspnoea, defined as moderate-to-severe dyspnoea corresponding to NYHA functional class ≥ II, was also more common among ever users (20.5% vs 9.1%; *P* < 0.05). Cardiac involvement followed the same pattern, with higher frequencies of arrhythmias requiring treatment, pericardial effusion, diastolic dysfunction, pulmonary hypertension, ventricular arrhythmias, conduction blocks and reduced left ventricular ejection fraction among ever users (all *P* < 0.05). As expected, GI involvement was markedly enriched among ever users, including oesophageal symptoms (85.1% vs 47.4%), gastric symptoms (43.4% vs 17.2%), intestinal symptoms (48.8% vs 28.4%) and malabsorption syndrome (6.0% vs 2.2%) (all *P* < 0.05).

Markers of systemic disease severity were also more frequent among ever users, including renal crisis, muscle weakness and atrophy, elevated CRP and proteinuria >300 mg/24 h (all *P* < 0.05). Finally, renal involvement was also more frequent among ever-PPI users, including higher rates of scleroderma renal crisis (3.0% vs 1.3%) and proteinuria >300 mg/24 h (14.6% vs 10.0%) (all *P* < 0.05).

### Mortality analysis

During follow-up, a total of 835 deaths (7.8%) were observed. Crude mortality was higher among patients ever exposed to PPIs compared with never users (8.6% vs 4.3%, *P* < 0.05). In unadjusted analyses, PPI exposure was associated with an increased risk of all-cause mortality (HR 2.75, 95% CI 2.09–3.62). In the IPTW-weighted time-varying Cox model, PPI exposure remained associated with higher mortality (HR 1.88, 95% CI 1.40–2.54; *P* < 0.005, Table 3). This estimate was consistent in sensitivity analyses applying a 6-month lag time (HR 1.75, 95% CI 1.30–2.35; *P* < 0.005) (Fig. 1a). There was no evidence of violation of the proportional hazard’s assumption for PPI exposure. Analyses stratified by follow-up tertiles showed a stronger association in the early period, whereas HRs were comparable and not statistically different in later intervals, with overlapping CIs. Additional adjustment for baseline csDMARD and/or bDMARD use did not materially change the association between PPI exposure and all-cause mortality (HR 1.90, 95% CI 1.41–2.55; *P* < 0.005). Baseline csDMARD/bDMARD use was not independently associated with mortality in this model (HR 1.20, 95% CI 0.94–1.53; *P* = 0.15) (Supplementary Table S3).

**Figure 1:**
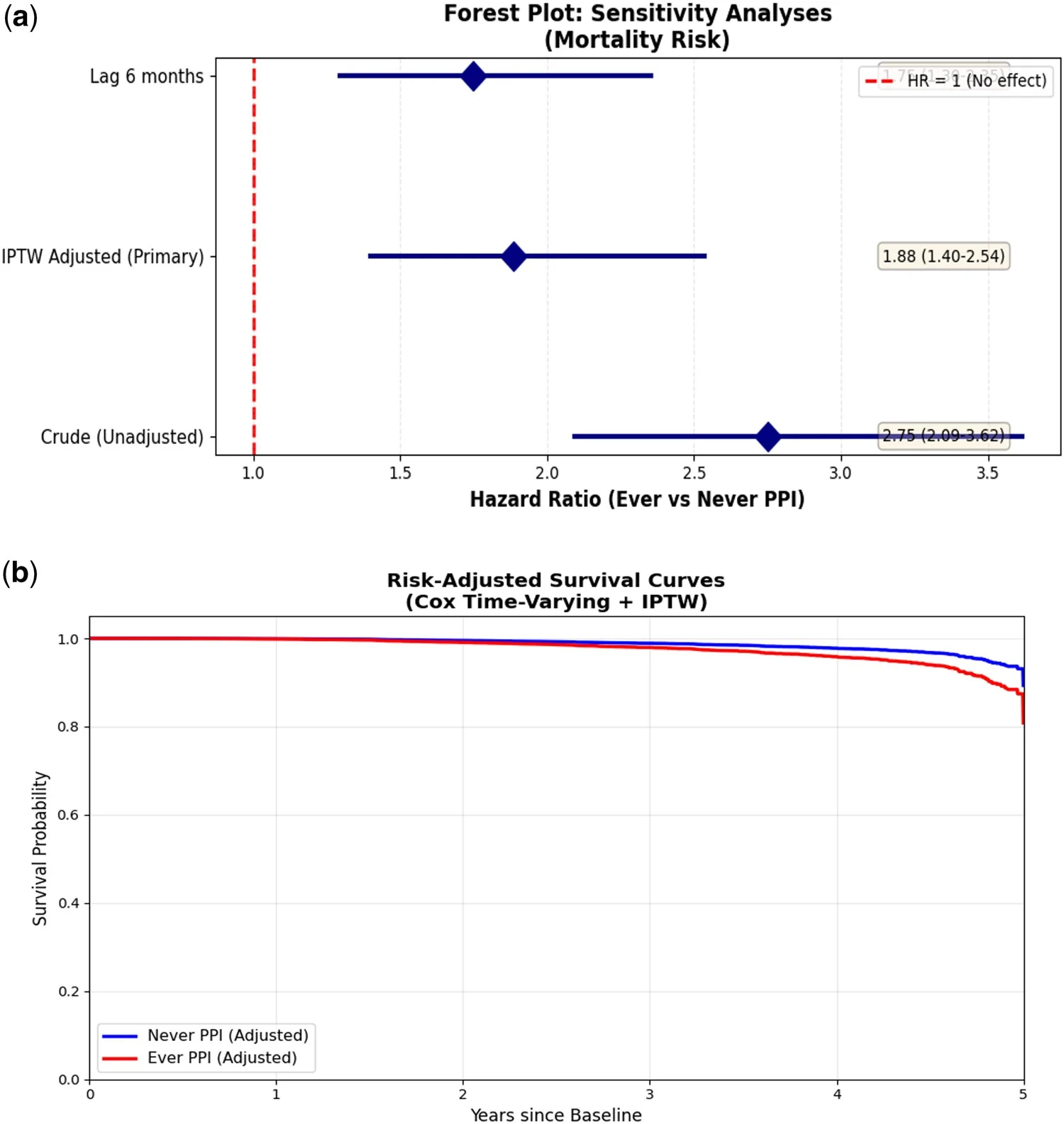
Association between PPI exposure and all-cause mortality. Panel **A** displays the results of analyses for all-cause mortality, including the unadjusted model, the primary IPTW-adjusted time-varying Cox model and the model applying a 6-month lag time. Hazard ratios are shown with 95% CIs. Panel **B** shows IPTW-adjusted survival curves derived from a time-varying Cox proportional hazards model, comparing patients ever exposed to PPIs with never users over a 5-year follow-up

**Table 3:** IPTW-adjusted time-varying Cox proportional hazards model for all-cause mortality.

	HR	95% CI		*P*-value
**PPI exposure**	1.88	1.40	2.54	**<0.005**
**Age**	1.06	1.05	1.07	**<0.005**
**Disease duration**	1.00	0.98	1.02	0.73
**Female sex**	0.63	0.48	0.82	**<0.005**
**Digital ulcers (ever)**	1.97	1.57	2.47	**<0.005**
**Proteinuria**	1.09	0.68	1.73	0.72
**Muscle weakness**	1.27	0.97	1.67	0.08
**LVEF <50%**	1.50	1.01	2.23	0.05
**dcSSc**	1.28	0.99	1.64	0.06
**Renal crisis**	1.69	0.93	3.05	0.08
**Prominent dyspnoea**	1.68	1.24	2.29	**<0.005**
**CRP elevation**	1.31	1.02	1.68	**<0.05**
**ILD**	1.05	0.83	1.33	0.67
**DLCO <60%**	1.53	1.19	1.98	**<0.005**
**FVC <70%**	1.59	1.19	2.11	**<0.005**
**Oesophageal symptoms**	0.88	0.70	1.10	0.25

Hazard ratios (HRs) and 95% CIs for the association between PPI exposure and all-cause mortality, estimated using a time-varying Cox proportional hazards model weighted by IPTW. The model was adjusted for established prognostic variables from the SCOpE score and presence of oesophageal symptoms. Hazard ratio for age is expressed per 1-year increase. Prominent dyspnoea was defined as moderate-to-severe dyspnoea corresponding to NYHA functional class ≥ II. *P*-values refer to two-sided tests. Statistically significant values (*P* < 0.05) are shown in bold.

dcSSc: diffuse cutaneous SSc; DLCO: diffusion lung capacity of carbon monoxide; FVC: forced vital capacity; ILD: interstitial lung disease; LVEF: left ventricular ejection fraction; PPI: proton pump inhibitors.

Adjusted survival curves derived from the IPTW-weighted model showed a modest but consistent separation over time, with slightly lower survival probabilities among PPI users (Fig. 1b). However, RMST at 5 years differed minimally between groups (4.94 years in never users vs 4.88 years in ever users; difference −0.05 years, ∼18 days), suggesting that the observed increase in relative mortality risk was not associated with a clinically meaningful reduction in short- to mid-term survival.

### Impact of PPI therapy on ILD progression

Among patients with established ILD at baseline (*n* = 4042), PPI exposure was highly prevalent (83.8% ever users and 16.2% never users), and a total of 403 progression events were observed for FVC decline ≥10%, 726 for FVC decline ≥5% and 457 for DLCO decline ≥10% during follow-up. In the primary IPTW-weighted time-varying Cox model, PPI exposure was not significantly associated with a reduced risk of severe functional progression defined as FVC decline ≥10% (HR 0.90, 95% CI 0.73–1.10; *P* = 0.30, Fig. 2a) (Table 4). Results were consistent after a 6-month lag (HR 0.87, 95% CI 0.71–1.08; *P* = 0.20; Fig. 2b). In contrast, when ILD progression was defined using moderate functional decline (FVC ≥5%), PPI exposure was associated with a significantly lower risk of progression (HR 0.76, 95% CI 0.65–0.88; *P* < 0.005) (Supplementary Fig. S1 and Supplementary Table S4). This association remained unchanged in sensitivity analyses applying a 6-month lag time (HR 0.74, 95% CI 0.64–0.87; *P* < 0.005). Similarly, PPI exposure was associated with a markedly lower risk of DLCO decline ≥10% (HR 0.57, 95% CI 0.47–0.69; *P* < 0.005), with highly consistent estimates across unadjusted, IPTW-adjusted and lag-time models (HR 0.56, 95% CI 0.46–0.68; *P* < 0.005) (Supplementary Fig. S2 and Supplementary Table S5). Additional adjustment for pulmonary hypertension did not materially change the results (HR 0.52, 95% CI 0.43–0.63; *P* < 0.005) (Supplementary Table S6).

**Figure 2:**
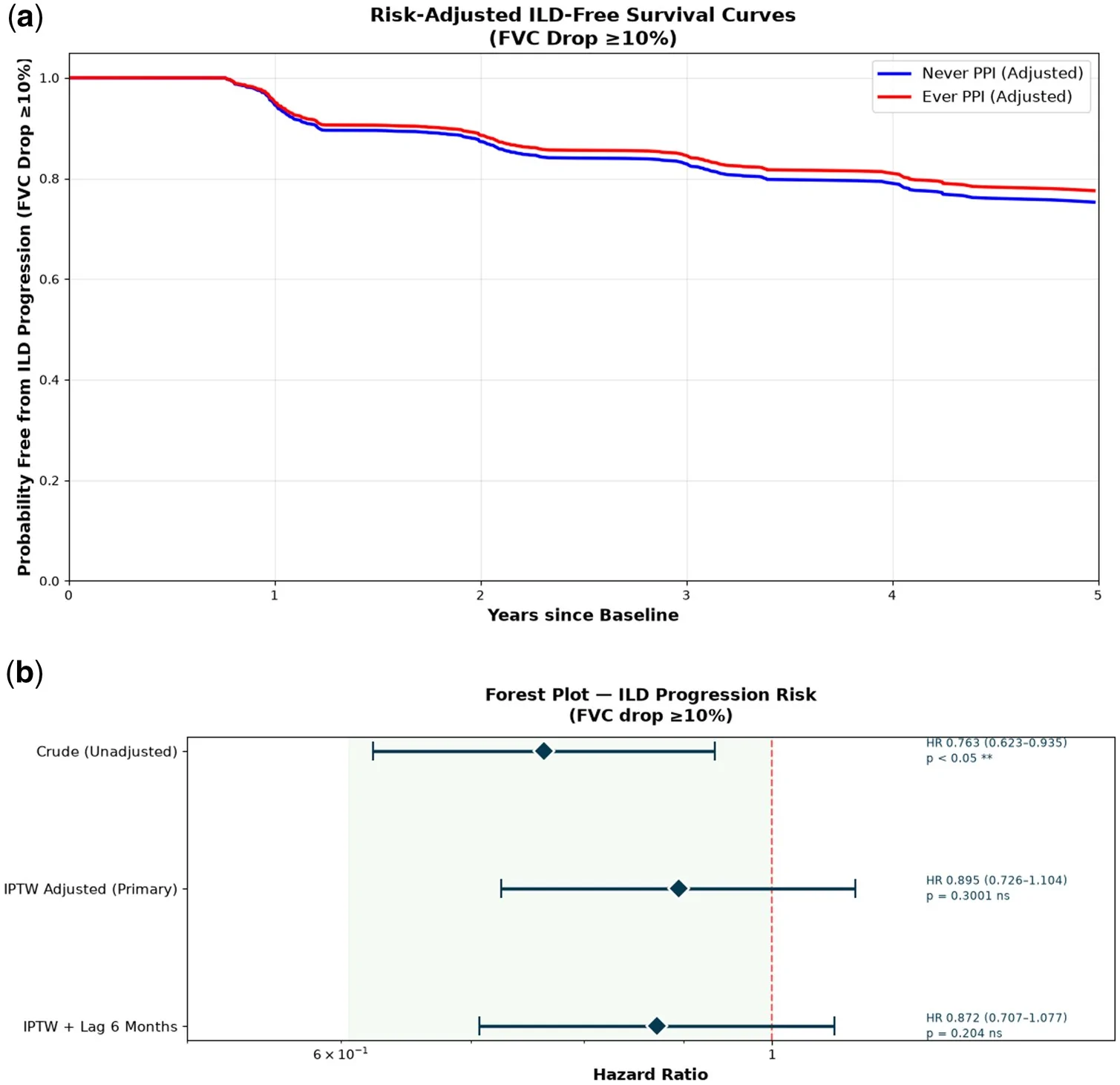
Association between PPI exposure and ILD progression defined by FVC decline ≥10%. Panel **A** shows IPTW-adjusted ILD-free survival curves derived from a time-varying Cox proportional hazards model, comparing patients ever exposed to PPIs with never users. ILD progression was defined as the first qualifying FVC decline ≥10% observed between two consecutive visits separated by approximately 1 year. Panel **B** presents hazard ratios with 95% CIs for ILD progression across different analytical approaches, including the unadjusted model, the primary IPTW-adjusted time-varying Cox model and the sensitivity analysis applying a 6-month lag time

**Table 4:** IPTW-adjusted time-varying Cox proportional hazards model for ILD progression defined by FVC decline ≥10%.

	HR	95% CI		*P*-value
**PPI exposure**	0.83	0.67	1.02	0.08
**Age**	1.00	1.00	1.01	0.25
**Disease duration**	0.99	0.97	1.01	0.26
**Erythrocyte sedimentation rate**	1.00	0.99	1.00	0.31
**%DLCO**	1.01	1.00	1.01	**<0.005**
**csDMARD/bDMARD treatment**	0.83	0.67	1.02	0.07
**mRSS**	1.00	0.99	1.02	0.73
**Female sex**	0.95	0.72	1.24	0.68
**Scl70 positivity**	1.06	0.85	1.32	0.62
**Presence of oesophageal symptoms**	0.89	0.72	1.10	0.29
**Prominent dyspnoea**	1.13	0.79	1.61	0.52
**dcSSc**	1.25	1.00	1.59	**0.05**

Hazard ratios (HRs) and 95% CIs for the association between PPI exposure and ILD progression, defined as a relative decline in FVC ≥10% from baseline. Estimates were obtained using a time-varying Cox proportional hazards model weighted by inverse probability of treatment (IPTW) and adjusted for demographic characteristics, disease duration, inflammatory markers, baseline pulmonary function, clinical manifestations and immunosuppressive treatment. Hazard ratio for age is expressed per 1-year increase. Prominent dyspnoea was defined as moderate-to-severe dyspnoea corresponding to NYHA functional class ≥ II. *P*-values refer to two-sided tests. Statistically significant values (*P* < 0.05) are shown in bold.

bDMARD: biologic DMARD; csDMARD: conventional synthetic DMARD; DLCO: diffusion lung capacity of carbon monoxide; dcSSc: diffuse cutaneous SSc; mRSS: modified Rodnan Skin Score; PPI: proton pump inhibitors.

RMST analyses over a 5-year horizon showed modest absolute differences. For FVC decline ≥10%, ever-PPI users remained free from progression for 4.11 years compared with 4.02 years among never users, ∼33 days difference. For FVC decline ≥5%, the corresponding estimates were 3.85 vs 3.56 years, with a difference of ∼3.5 months. For DLCO decline ≥10%, ever-PPI users remained free from progression for 4.30 years compared with 3.88 years among never users, corresponding to a difference of ∼5 months.

Diagnostics suggested time-dependence in the association between PPI exposure and ILD progression endpoints. Therefore, HRs from the time-varying Cox models were interpreted as average associations over follow-up and complemented by RMST analyses to quantify absolute progression-free differences over a fixed 5-year horizon.

## Discussion

In this large multinational real-world analysis from the EUSTAR registry, PPI therapy was highly prevalent and predominantly prescribed in patients with a more severe multisystem disease phenotype. Although PPI exposure was associated with higher relative mortality estimates, absolute survival differences over 5 years were minimal and only modest signals on protection against ILD functional progression were observed.

In our cohort, patients receiving PPIs exhibited a greater burden of systemic disease, including more severe GI, vascular, cardiac and renal involvement, a higher prevalence of ILD with poorer pulmonary function and more intensive background therapy. This pattern strongly supports confounding by indication, suggesting that PPI exposure primarily identifies patients with a more severe SSc phenotype rather than providing evidence of a direct harmful effect of PPI therapy. Previous studies have similarly shown that severe GI and oesophageal involvement in SSc tends to cluster with diffuse cutaneous disease, digital ulcers, impaired DLCO and overall poorer prognosis [24, 25]. Physiological studies have demonstrated that severe oesophageal dysmotility is frequently associated with more advanced systemic disease and worse pulmonary function [26, 27]. In this context, PPI exposure likely represents a surrogate marker of severe oesophageal involvement within a broader aggressive disease phenotype. Similarly, the higher frequency of renal manifestations among ever-PPI users, including scleroderma renal crisis and proteinuria, should be interpreted as part of the broader multisystem disease-severity profile associated with PPI exposure. As renal involvement was assessed descriptively using a worst-ever approach and not modelled as an incident outcome, these findings should not be interpreted as a PPI-associated renal safety signal.

This clinical context is important for interpreting the mortality findings. Although PPI exposure was associated with higher all-cause mortality in relative terms, absolute survival differences over 5 years were minimal, corresponding to approximately only 20 days. Previous studies have reported different results. In particular, an analysis from the German Network for SSc reported improved survival among PPI-treated patients with SSc-ILD [28], while the Australian Scleroderma Cohort Study also observed improved survival in patients receiving anti-reflux therapy [29]. These discrepancies likely reflect differences in study populations and analytical approaches. Both the German and Australian cohorts focused specifically on patients with established SSc-ILD, whereas our analysis included a broader SSc population. In addition, earlier studies relied on static exposure definitions and limited adjustment for treatment dynamics. By modelling PPI exposure as a time-varying variable within a large multinational registry, our study provides a complementary perspective on long-term treatment associations in routine clinical practice.

Beyond survival, the relationship between anti-reflux therapy and pulmonary outcomes in SSc has also been explored in several observational cohorts with heterogeneous results. In our analysis, PPI exposure was associated with only modest signals on pulmonary functional trajectories, with a small attenuation of DLCO decline translating into limited progression-free time and no measurable survival benefit. Previous studies have reported variable findings. While the German Network cohort suggested improved progression-free survival among PPI-treated patients with SSc-ILD [28], several other observational cohorts did not demonstrate a protective effect of acid-suppressive therapy on lung disease. Analyses from the Canadian Scleroderma Research Group did not show a reduced risk of incident ILD among SSc patients receiving gastroprotective agents [29], and data from the CONQUER registry and clinical trial-derived cohorts suggest that reflux severity, rather than PPI therapy itself, is associated with pulmonary function decline and ILD progression [30, 31]. Together, these findings indicate that the relationship between reflux, acid suppression and lung disease in SSc is complex and influenced by differences in study design and patient populations.

From a mechanistic perspective, the limited pulmonary impact observed in our study is biologically plausible. Although PPIs effectively reduce gastric acidity, they do not address the oesophageal dysmotility, reflux volume, non-acid reflux material or extra-oesophageal GI dysmotility [32, 33] that impact refractory GERD and characterize GI involvement in SSc. Indeed, in our study, non-ever-PPI users were much less likely to receive treatment with promotility agents, suggesting that their reflux disease is overall less severe and/or driven (at least partially) through failure of motility mechanisms. Therefore, reflux volume and non-acid reflux material may persist despite acid suppression. Physiological studies have shown that abnormal oesophageal acid exposure frequently persists even during high-dose PPI therapy, and that weakly acidic or non-acid reflux is common in SSc patients [5, 34, 35]. Accordingly, acid suppression alone is unlikely to substantially modify pulmonary disease trajectories in SSc; and increases the therapeutic rationale for targeting motility-based pharmacological mechanisms [36, 37]. Furthermore, in this context, subsets of patients with more extensive GI dysfunctions (e.g. combinations of oesophageal dysmotility plus weak LES plus gastroparesis) may be at higher risk for microaspiration and related ILD progression, though this study was not designed to differentiate these subgroups.

Our study has several strengths, including the large sample size of the EUSTAR registry, and the use of analytical approaches specifically designed for real-world data. Modelling PPI exposure as a time-varying variable, combined with IPTW, allowed us to better account for changes in treatment over time and to mitigate immortal-time bias and confounding by indication. The use of multiple definitions of ILD progression and complementary absolute measures, such as RMST, further strengthens the robustness and clinical interpretability of our findings. However, this study also has limitations inherent to observational registry-based analyses. Detailed information on PPI dose, specific compound, adherence, treatment indication and cumulative treatment intensity was not available. In addition, H2 receptor antagonists, alginates, antacids and other non-PPI anti-reflux therapies were not systematically captured; therefore, some patients classified as never exposed to PPIs may have received alternative acid-suppressive or anti-reflux treatment, potentially diluting exposure contrasts. Objective upper GI assessments, including endoscopy and endoscopic findings such as oesophagitis, strictures or Barrett’s oesophagus, were also not systematically available. As a result, we could not distinguish whether PPI exposure reflected symptomatic GERD treatment, objectively confirmed reflux severity or broader physician prescribing patterns across centres. Furthermore, residual confounding cannot be completely excluded. ILD progression was assessed using pulmonary function parameters without systematic radiologic evaluation. Finally, although exclusion of patients with a single recorded visit followed by death could theoretically introduce selection bias in the mortality analysis, only five patients met this criterion, making a material impact on the estimates unlikely.

In conclusion, PPI use is highly prevalent in patients with SSc and clusters with a more severe disease phenotype. In this large real-life SSc cohort, although associated with higher all-cause mortality, absolute survival differences were minimal and likely reflect underlying disease severity. Only modest attenuation of ILD progression was observed, and with limited clinical utility. These findings support continued PPI use for symptomatic GERD while a careful, individualized reassessment of long-term therapy is warranted.

## Supplementary Material

keag521_Supplementary_Data

## Data Availability

Data are available for research purposes upon request.
