# Supplementary material for: Proton pump inhibitor use and long-term outcomes in SSc: a real-life analysis from the EUSTAR database
Source: Rheumatology (Oxford). 2026 Sep 18;65(10):keag521. doi: 10.1093/rheumatology/keag521 (PMC13637600; doi:10.1093/rheumatology/keag521)
Supplement: Supplementary file 1 [file keag521_supplementary_data.zip › rhe-26-1166-File004.docx]

List of EUSTAR Collaborators

| Ulrich Andreas Walker | Basel (Switzerland) |
| --- | --- |
| Radim Bečvář | Prague (Czech Republic) |
| Elise Siegert | Berlin (Germany) |
| Yannick Allanore | Paris (France) |
| Carlomaurizio Montecucco | Pavia (Italy) |
| Murat Inanc | Istanbul (Turkey) |
| Patricia Carreira | Madrid (Spain) |
| Gábor Kumánovics | Pecs (Hungary) |
| Michele Iudici | Geneva (Switzerland) |
| Przemyslaw Kotyla | Katowice (Poland) |
| Elisabetta Zanatta | Padova (Italy) |
| Gianluca Moroncini | Ancona (Italy) |
| Paolo Airò | Brescia (Italy) |
| Alexandra Balbir-Gurman | Haifa (Israel) |
| Annamaria Iagnocco | Torino (Italy) |
| Josko Mitrovic | Zagreb (Croatia) |
| Christopher Denton | London (UK) |
| Madelon Vonk | Nijmegen (The Netherlands) |
| Gianluigi Bajocchi | Reggio Emilia (Italy) |
| Tânia Santiago | Coimbra (Portugal) |
| Maria De Santis | Rozzano, Milano (Italy) |
| David Launay | Lille (France) |
| Valeria Riccieri | Roma (Italy) |
| Andra Balanescu | Bucharest (Romania) |
| Ana Maria Gheorghiu | Bucharest (Romania) |
| Vanessa Smith | Gent (Belgium) |
| Addolorata Corrado | Foggia (Italy) |
| Mette Mogensen | Copenhagen (Denmark) |
| Marie Vanthuyne | Brussels (Belgium) |
| Juan Jose Alegre Sancho | Valencia (Spain) |
| Branimir Anić | Zagreb (Croatia) |
| Carolina de Souza Müller | Curitiba (Brazil) |
| Alberto Cauli | Monserrato (Italy) |
| Kamal Solanki | Hamilton (New Zealand) |
| Eiman Soliman | Alexandria (Egypt) |
| Edoardo Rosato | Roma (Italy) |
| Britta Maurer | Bern (Switzerland) |
| Sophie Blaise | Grenoble (France) |
| Patricia Senet | Paris (France) |
| Emmanuel Chatelus | Strasbourg (France) |
| Francesco Del Galdo | Leeds (United Kingdom) |
| Ivan Castellví | Barcelona (Spain) |
| Massimiliano Limonta | Bergamo (Italy) |
| Antonella Marcoccia | Roma (Italy) |
| Ina Koetter | Bad Bramstedt (Germany) |
| Vivien Hsu | New Brunswick (USA) |
| Dinesh Khanna | Ann Arbor, Michigan (USA) |
| Carolina Teles | Coimbra (Portugal) |
| Yair Levy | kfar-saba (Israel) |
| Elena Rezus | Iasi (Romania) |
| Daniel Brito de Araujo | Pelotas (Brazil) |
| Giovanna Cuomo | Naples (Italy) |
| Petros Sfikakis | Athens (Greece) |
| Ana-Maria Ramazan | Constanta City (Romania) |
| Jeska de Vries-Bouwstra | Leiden (The Netherlands) |
| Alain Lescoat | Rennes (France) |
| Julia Spierings | Utrecht (The Netherlands) |
| Fabiola Atzeni | Messina (Italy) |
| Masataka Kuwana | Tokyo (Japan) |
| Mickaël Martin | Poitiers (France) |
| Alfredo Guillén-Del-Castillo | Barcelona (Spain) |
| Magda Parvu | Bucharest (Romania) |
| Gonçalo Boleto | Lisboa (Portugal) |
| Nicoletta Del Papa | Milano (Italy) |
| Enrico Selvi | Siena (Italy) |
| Marta Mosca | Pisa (Italy) |
| Ulrich Gerth | Rheinfelden (Switzerland) |
| Duygu Temiz Karadag | Kocaeli (Turkey) |
| Anastas Batalov | Plovdiv (Bulgaria) |
| Cristina Maglio | Gothenburg (Sweden) |
| Miriam Retuerto | Leon (Spain) |
| Monique Hinchcliff | New Haven (USA) |
| Samar Tharwat | Dakahlia (Egypt) |
| Ana Cristina Cordeiro | Almada (Portugal) |
| Francesco Benvenuti | Vicenza (Italy) |
| Andrea-Hermina Györfi | Düsseldorf (Germany) |
| Lilian Maria Lopez Nunez | Palma de Mallorca (Spain) |
| Rossella De Angelis | Marche (Italy) |
| Alejandro Brigante | Buenos Aires (Argentina) |
| Yasser El Miedany | Cairo (Egypt) |
| Amato de Paulis | Naples (Italy) |
| Chris Derk | Philadelphia (USA) |
| Lijun Zhang | Hong-Kong (China) |
| Bogdan Batko | Cracow (Poland) |
| Ivette Casafont Sole | Barcelona (Spain) |
| Anna Lewandowska-Polak | Lodz (Poland) |
| Belén Atienza-Mateo | Santander (Spain) |
| Tuncay Duruöz | Istanbul (Turkey) |
| Seda Colak | Ankara (Turkey) |
| Janeth Villegas Guzmán | Lima (Chile) |
| Claudia Mora-Trujillo | Lima (Chile) |
| Boris Lindner | Vienna (Austria) |
| Maria Sole Chimenti | Rome (Italy) |
| Samah A. El-Bakry | Cairo (Egypt) |
| Vera Ortiz-Santamaria | Barcelona (Spain) |
| Jorge Rojas-Serran | (Mexico) |
| Fatma Alibaz-Oner | Istanbul (Turkey) |
| Emine Duygu Ersözlü | Adana (Turkey) |
| Roberto Muñoz Louis | Santo Domingo D.N (Dominican Republic) |
| Minrui Liang | Shanghai (China) |
| Massimo Triggiani | Salerno (Italy) |
| Anastasios Karamanakos | Athens (Greece) |
| Bianca Dumitru | Coventry (UK) |
| Lucy Carter | Newcastle (UK) |
| Beatrice André | Liege (Belgium) |
| Giuseppe Murdaca | Sarzana (Italy) |
| Patricia Corzo Garcia | Barcelona (Spain) |
| Theodora Simopoulou | Larissa (Greece) |
| Fabio Cacciapaglia | Acquaviva delle Fonti, Bari (Italy) |
| Tejaswini Ramineni | Hyderabad (India) |
| Gaia Mansuco | Ticino (Switzerland) |
| Jhonny Calizaya-Estrada | Arequipa (Peru) |
| Gehad Gamal Maghraby | Cairo (Egypt) |
| Mustafa Erdogan | Istanbul (Turkey) |
| Wen-Nan Huang | Taichung (Taiwan) |
| Graça Sequeira | Faro (Portugal) |
| Greta Pellegrino | Milan (Italy) |
| Cláudia Vaz | Guarda (Portugal) |
| Maya Buch | Manchester (UK) |
| Joanna Kosałka-Węgiel | Kraków (Poland) |
