# Supplementary material for: Proton pump inhibitor use and long-term outcomes in SSc: a real-life analysis from the EUSTAR database
Source: Rheumatology (Oxford). 2026 Sep 18;65(10):keag521. doi: 10.1093/rheumatology/keag521 (PMC13637600; doi:10.1093/rheumatology/keag521)
Supplement: Supplementary file 1 [file keag521_supplementary_data.zip › rhe-26-1166-File005.docx]

**Table S1. Covariates included in the propensity score model for IPTW weighting for all-cause mortality.**

IPTW, inverse probability of treatment weighting; LVEF, left ventricular ejection fraction; dcSSc, diffuse cutaneous systemic sclerosis; CRP, C-reactive protein; ILD, interstitial lung disease; DLCO, diffusing capacity for carbon monoxide; FVC, forced vital capacity; NYHA, New York Heart Association.

| **Variable** | **Type** | **Definition** |
| --- | --- | --- |
| Age | Continuous | Age at baseline (years) |
| Disease duration | Continuous | Disease duration at baseline |
| Female sex | Binary | Female vs male |
| Digital ulcers (ever) | Binary | History of digital ulcers |
| Proteinuria | Binary | Presence of proteinuria |
| Muscle weakness | Binary | Presence of muscle weakness |
| LVEF <50% | Binary | Left ventricular ejection fraction <50% |
| dcSSc | Binary | Diffuse cutaneous systemic sclerosis |
| Renal crisis | Binary | History of scleroderma renal crisis |
| Prominent dyspnea | Binary | Moderate-to-severe dyspnea corresponding to NYHA functional class ≥II |
| CRP elevation | Binary | Elevated C-reactive protein |
| ILD | Binary | Presence of interstitial lung disease |
| DLCO <60% | Binary | Diffusing capacity for carbon monoxide <60% predicted |
| FVC <70% | Binary | Forced vital capacity <70% predicted |
| Esophageal symptoms | Binary | Presence of dysphagia and/or reflux symptoms |

**Table S2. Covariates included in the propensity score model for IPTW weighting for ILD progression analyses.**

Abbreviations: IPTW, inverse probability of treatment weighting; ILD, interstitial lung disease; FVC, forced vital capacity; csDMARD, conventional synthetic disease-modifying antirheumatic drug; bDMARD, biologic disease-modifying antirheumatic drug; SSc, systemic sclerosis; NYHA, New York Heart Association.

| **Variable** | **Type** | **Definition** |
| --- | --- | --- |
| Age | Continuous | Age at the start of each interval, in years |
| Disease duration | Continuous | Disease duration at the start of each interval, in years |
| Erythrocyte sedimentation rate | Continuous | Erythrocyte sedimentation rate measured at the start of each interval or at the preceding visit |
| FVC (% predicted) | Continuous | Forced vital capacity, % predicted, measured at the start of each interval or at the preceding visit |
| csDMARD and/or bDMARD use | Binary | Current use of conventional synthetic and/or biologic disease-modifying antirheumatic drugs at the start of each interval or at the preceding visit |
| Modified Rodnan Skin Score | Continuous | Modified Rodnan Skin Score measured at the start of each interval or at the preceding visit |
| Female sex | Binary | Female vs male |
| Anti-Scl70 positivity | Binary | Positive anti-topoisomerase I antibody status |
| Esophageal symptoms (dysphagia/reflux) | Binary | Presence of dysphagia and/or reflux symptoms at the start of each interval or at the preceding visit |
| Prominent dyspnea | Binary | Moderate-to-severe dyspnea, corresponding to NYHA functional class ≥II, at the start of each interval or at the preceding visit |
| Diffuse cutaneous SSc | Binary | Diffuse cutaneous systemic sclerosis versus other subsets |

**Table S3. Sensitivity analysis for all-cause mortality additionally adjusted for baseline csDMARD/bDMARD use.** Hazard ratios (HRs) and 95% confidence intervals (CIs) were estimated using an IPTW-weighted time-varying Cox proportional hazards model. P-values refer to two-sided tests.

Abbreviations: bDMARD, biologic disease-modifying antirheumatic drug; csDMARD, conventional synthetic disease-modifying antirheumatic drug; CRP, C-reactive protein; DLCO, diffusing capacity of the lung for carbon monoxide; FVC, forced vital capacity; LVEF, left ventricular ejection fraction; PPI, proton pump inhibitor.

| **Variable** | **HR** | **95% CI** | **p value** |
| --- | --- | --- | --- |
| **PPI exposure** | **1.90** | **1.41–2.55** | **<0.005** |
| Age | 1.06 | 1.05–1.07 | **<0.005** |
| Disease duration | 1.00 | 0.98–1.01 | 0.65 |
| Female sex | 0.62 | 0.48–0.82 | **<0.005** |
| Digital ulcers | 1.97 | 1.57–2.47 | **<0.005** |
| Proteinuria | 1.11 | 0.70–1.76 | 0.67 |
| Muscle weakness | 1.27 | 0.96–1.67 | 0.09 |
| LVEF <50% | 1.50 | 1.01–2.24 | **<0.05** |
| Diffuse cutaneous disease | 1.28 | 1.00–1.65 | **0.05** |
| Renal crisis | 1.67 | 0.92–3.01 | 0.09 |
| Prominent dyspnea | 1.66 | 1.22–2.26 | **<0.005** |
| CRP elevation | 1.31 | 1.02–1.68 | **<0.05** |
| Interstitial lung disease | 1.03 | 0.82–1.30 | 0.80 |
| DLCO <60% predicted | 1.53 | 1.18–1.97 | **<0.005** |
| FVC <70% predicted | 1.59 | 1.19–2.11 | **<0.005** |
| Esophageal symptoms (dysphagia/reflux) | 0.88 | 0.70–1.10 | 0.25 |
| csDMARD/bDMARD use | 1.20 | 0.94–1.53 | 0.15 |

**Figure S1. Risk-adjusted ILD-free survival curves according to PPI exposure (FVC decline ≥5%).**

Adjusted survival curves derived from the IPTW-weighted time-varying Cox model comparing patients ever exposed to PPI with never users. The analysis shows a higher probability of remaining free from ILD progression among PPI users over time, although absolute differences were modest.

**Table S4. IPTW-adjusted time-varying Cox proportional hazards model for ILD progression defined as FVC decline ≥5%.**

Hazard ratios were estimated using IPTW-weighted time-varying Cox proportional hazards models.

| **Variable** | **HR** | **95% CI** | **p value** |
| --- | --- | --- | --- |
| PPI exposure | 0.76 | 0.65–0.88 | **<0.005** |
| Disease duration | 0.99 | 0.98–1.00 | 0.135 |
| Erythrocyte sedimentation rate | 1.00 | 1.00–1.01 | 0.879 |
| DLCO % predicted | 1.01 | 1.00–1.01 | **<0.005** |
| csDMARD/bDMARD use | 0.83 | 0.71–0.97 | **<0.05** |
| Modified Rodnan Skin Score | 1.01 | 0.99–1.02 | 0.353 |
| Female sex | 0.87 | 0.72–1.06 | 0.164 |
| Scl-70 positivity | 1.07 | 0.92–1.25 | 0.397 |
| Esophageal symptoms | 0.96 | 0.83–1.12 | 0.634 |
| Prominent dyspnea | 1.19 | 0.90–1.57 | 0.229 |
| Limited cutaneous SSc | 0.98 | 0.83–1.16 | 0.808 |

**Figure S2. Risk-adjusted ILD-free survival curves according to PPI exposure (DLCO decline ≥10%).**

Adjusted survival curves derived from the IPTW-weighted time-varying Cox proportional hazards model comparing patients ever exposed to PPI with never users. The analysis demonstrates a consistently higher probability of remaining free from ILD progression among PPI users over time, with modest but persistent separation between groups.

**Table S5. IPTW-weighted time-varying Cox model for ILD progression defined as DLCO decline ≥10%.**

Hazard ratios were estimated using IPTW-weighted time-varying Cox proportional hazards models.

| **Variable** | **HR** | **95% CI** | **p value** |
| --- | --- | --- | --- |
| **PPI exposure** | **0.57** | **0.47–0.69** | **<0.005** |
| Disease duration | 0.98 | 0.97–1.00 | **<0.05** |
| Erythrocyte sedimentation rate | 1.00 | 0.99–1.01 | 0.946 |
| FVC % predicted | 1.01 | 1.01–1.02 | **<0.005** |
| csDMARD/bDMARD use | 0.58 | 0.47–0.71 | **<0.005** |
| Modified Rodnan Skin Score | 1.00 | 0.99–1.02 | 0.669 |
| Female sex | 0.64 | 0.51–0.81 | **<0.005** |
| Scl-70 positivity | 1.30 | 1.07–1.59 | **<0.005** |
| Esophageal symptoms (dysphagia/reflux) | 0.94 | 0.77–1.14 | 0.533 |
| Prominent dyspnea | 1.08 | 0.73–1.59 | 0.706 |
| Limited cutaneous SSc | 1.09 | 0.88–1.35 | 0.442 |

**Table S6. IPTW-weighted time-varying Cox model for ILD progression defined as DLCO decline ≥10%, with additional adjustment for pulmonary hypertension.**

Hazard ratios were estimated using IPTW-weighted time-varying Cox proportional hazards models.

| **Variable** | **HR** | **95% CI** | **p value** |
| --- | --- | --- | --- |
| **PPI exposure** | **0.52** | **0.43–0.63** | **<0.005** |
| Age | 1.00 | 0.99–1.01 | 0.88 |
| Disease duration | 0.98 | 0.97–1.00 | 0.06 |
| Erythrocyte sedimentation rate | 1.00 | 0.99–1.00 | 0.70 |
| FVC (% predicted) | 1.01 | 1.01–1.02 | **<0.005** |
| csDMARD and/or bDMARD use | 0.90 | 0.74–1.10 | 0.32 |
| Modified Rodnan Skin Score | 1.00 | 0.99–1.02 | 0.72 |
| Female sex | 0.66 | 0.52–0.85 | **<0.005** |
| Anti-Scl70 positivity | 1.21 | 0.98–1.48 | 0.07 |
| Esophageal symptoms (dysphagia/reflux) | 0.98 | 0.80–1.19 | 0.84 |
| Prominent dyspnea | 0.95 | 0.63–1.42 | 0.79 |
| Diffuse cutaneous SSc (vs others) | 1.10 | 0.88–1.36 | 0.41 |
| Pulmonary hypertension | 1.12 | 0.79–1.59 | 0.52 |
